# Annotations

**Published:** 1903-02-07

**Authors:** 


### ANNOTATIONS.

### Human and Bovine Tuberculosis.

A NEW aspect of the tuberculosis problem is propounded by Dr. Nathan Raw, which, however, does not by any means diminish its complexity. On visiting the laboratories of Professor Koch in Berlin some months ago, he was much impressed with the distinctive cultural peculiarities of human and bovine tuberculosis, and he appears to have been led to fall in with the view which was again last year reiterated by Professor Koch, namely, that the two diseases and bovine tuberculosis, or *Perlsucht*, and human tuberculosis as usually met with in the lungs, are quite distinct and separate. When, however, Professor Koch goes further and says that bovine tuberculosis cannot be conveyed to the human then Dr. Raw separates from him, holding that there is some evidence to show that there are two distinct varieties of tuberculosis affecting the human body, the one produced by human tubercle, and the other by bovine tubercle. He has lately met with two cases—one of tabes mesenterica, and the other of strumous knee-joint—cultures from both of which corresponded exactly with those from bovine tuberculosis or *Perlsucht*. Hence, although it may be true as stated by Koch that human and bovine tuberculosis are separate and distinct diseases, the deduction that therefore we need not trouble about the bovine form, in our efforts to prevent tuberculosis in man, may not be true at all. Dr. Raw suggests that while the true human tuberculosis is conveyed from one person to another by dust infection, generally from advanced cases of phthisis, bovine tuberculosis on the other hand, entering the alimentary canal in milk, may set up tabes mesenterica, and is probably the cause of enlarged lymph glands, tuberculous joints, and lupus. There is no doubt that many clinical observations suggest that tabes mesenterica and certain diseases of the joints generally regarded as tuberculous, stand somewhat apart from the common tuberculosis of consumption, and if Dr. Raw's hypothesis should prove correct much would be explained. Nevertheless the milk must still be boiled, and every effort must still be made to stamp out tuberculosis in cattle.

### A Lesson from Colney Hatch.

IT is impossible to pass over such an event as the appalling catastrophe at Colney Hatch without drawing attention to the important light it throws upon the danger to which the inmates of temporary hospitals constructed of inflammable materials are exposed. At the present time some note of warning of the danger incurred in the use of such buildings is particularly necessary in view of the fact that the spread of small-pox in many parts of the provinces is making it necessary, sometimes under conditions of urgency, hurry and excitement, to provide hospital accommodation for patients who cannot, in the present ill-vaccinated condition of the country, be safely treated in existing institutions. Under such circumstances a recourse to temporary structures is almost a foregone conclusion, and without doubt the most convenient material, and that which can be brought into use with the greatest rapidity, is corrugated iron with matchboard lining. There is much to be said for buildings so constructed; but they are horribly inflammable, and when once set on fire the dangers connected with them are manifold. Their convenience however and the rapidity with which they can be erected has led to their employment by many sanitary authorities. At Tottenham, during a more or less artificial scare about scarlet fever some years ago, a great hospital for 500 patients arose in the course of a few weeks; at Lower Tooting, again, the same thing happened, another great fever hospital springing into being, as at the waving of a magician's wand, to meet a demand for increased accommodation; while in the neighbourhood of the small-pox ships a large amount of accommodation has lately been provided. These buildings are constructed of the most inflammable materials, and are a constant source of anger. Meanwhile temporary hospitals are being erected all over the country. A temporary hospital however is but too often a desperately inflammable one, and this fact should not be forgotten by those who are responsible for the safety of the patients. Surely it is not too much to ask our architects to invent some means of erecting non-inflammable buildings for temporary purposes which shall not be inordinately expensive, and from which all wood shall be excluded.

### Hygiene in the Barber's Shop.

IT is a curious circumstance that, at the very moment that the medical papers in America are busying themselves with the danger of transferring contagious disease by the use of the razor, one of our contemporaries on this side of the water is carefully explaining to its readers why razor cuts are so innocuous. It is doubtless true that the cuts so often inflicted by the razor are seldom followed by serious consequences, and that this is largely due to the fact that the razor is more or less sterilised by immersion in hot water, and that the soap with which the face is freely lathered is an antiseptic. Thus, in ordinary parlance, a wound inflicted under such circumstances is a "clean cut," and that it should usually heal up without further difficulty is not by any means surprising. But although soap is an admirable disinfectant as against pus-producing organisms, and is in fact largely used in surgery for the purpose, we may fairly express some doubt as to its efficacy against such an infection as that of syphilis. Cases of the infection of razor cuts by this disease are reported with sufficient frequency to make it clear that we cannot afford to entirely neglect the dangers of the barber's shop. True it may not always be by the razor that the infection is inserted, but there are other things besides razors to be thought of. We say nothing of the brush, or even of the fingers, on which the soap may be imagined to exercise such influence as it possesses; but although the skin may be sterile and the razor may be clean, there is a certain sponge which some barbers delight in using which seems to us to have a very suspicious look, and there is a "puff" which undoubtedly goes from face to face without the slightest discrimination. Other countries are ahead of us in this matter. In a recent American paper we see it reported that an Italian barber in Buffalo was fined at the Municipal Court for neglecting the rules of cleanliness in wiping the faces of two customers with a single towel. This shows progress

